# APOE‐relevant tau oligomer polymorphs differentially impair synaptic functioning

**DOI:** 10.1002/alz70855_106052

**Published:** 2025-12-24

**Authors:** Naomi Moreno, Nikita Shchankin, Nemil Bhatt, Md Anzarul Haque, Agenor Limon, Rakez Kayed

**Affiliations:** ^1^ University of Texas Medical Branch, Galveston, TX, USA

## Abstract

**Background:**

Apolipoprotein E (APOE), the strongest genetic risk factor for late‐onset Alzheimer's disease, exist as 3 isoforms (APOE2, 3, 4) which differentially influence Alzheimer's disease risk and tau pathology in disease. Recent studies have revealed the polymorphic nature of amyloidogenic proteins, including tau, across different diseases, yet there remains a gap in knowledge concerning tau polymorphism across genetic variants such as the APOE isoforms. Here, we address this gap in knowledge by characterizing tau oligomers associated with each APOE isoform.

**Method:**

Brain‐derived tau oligomers and fibrils were isolated from tissue of patients with various APOE genotypes. Tau oligomers were characterized using proteinase K (PK) digestion and liquid chromatography‐tandem mass spectrometry (LC‐MS/MS) to investigate proteolytic stability and cleavage site accessibility to PK. Electrophysiology was used to investigate synaptotoxicity of the tau oligomers.

**Result:**

Tau oligomers differ in proteolytic stability and cleavage site profiles across the APOE isoforms, indicating conformationally‐distinct tau oligomer polymorphs. The tau oligomer polymorphs differentially impair synaptic functioning in an APOE isoform‐specific manner, with APOE4‐relevant tau oligomers inducing the strongest impairment of synaptic functioning.

**Conclusion:**

The APOE isoforms correspond with distinct tau oligomer polymorphs with varying synaptotoxicity. These findings highlight the need for APOE isoform to be considered when generating tau‐based therapies for Alzheimer's disease. Specific targeting of APOE isoform‐specific tau oligomer polymorphs could provide a novel method of mitigating Alzheimer's Disease pathology and combating disease progression.

